# Seroprevalence of hepatitis C antibodies in pregnant women in the state of Paraná, Brazil

**DOI:** 10.1016/j.clinsp.2026.100978

**Published:** 2026-04-28

**Authors:** Gabriele Aquery, Newton S. de Carvalho

**Affiliations:** Postgraduate Program in Obstetrics, Gynecology and Women's Health, Universidade Federal do Paraná, Curitiba, Brazil

**Keywords:** Hepatitis C Virus, Prenatal screening, Seroepidemiology, Vertical transmission, Pregnancy, Brazil, Public health, Prevalence Study, Low-Resource Setting, Antiviral Therapy

## Abstract

**Introduction:**

Hepatitis C (HCV) is an important cause of global morbidity and mortality and can progress to cirrhosis and hepatocellular carcinoma. Vertical transmission occurs in around 5%‒6% of cases, increasing in pregnant women with a high viral load or co-infection with HIV. Although universal screening is recommended by international guidelines, its implementation in Brazil is uneven. This study estimated the seroprevalence of anti-HCV antibodies among pregnant women attending public maternity hospitals in Paraná and compared the results with national data.

**Material and methods:**

This was an observational, descriptive, and cross-sectional population-based study to assess seroprevalence. The study included 1202 pregnant women aged between 16 and 49, treated in 14 public maternity hospitals in Paraná, in different regions of the state. Anti-HCV screening was carried out by ELISA, and reactive cases were confirmed by viral load (PCR). Sociodemographic and obstetric data and risk factors were obtained from questionnaires and medical records.

**Results:**

Nine participants tested positive for anti-HCV antibodies, corresponding to a seroprevalence of 0.75% (95% CI 0.34%‒1.40%). This seroprevalence falls within the range reported in previous Brazilian cross-sectional studies of pregnant women (0.06%–2.66%). No active infection was identified, as all reactive samples were HCV RNA-negative. More than half were unaware of their previous infection. No significant associations with traditional risk factors were observed.

**Discussion:**

The low anti-HCV seroprevalence and the absence of detectable viremia indicate a minimal risk of vertical transmission in the cohort studied. These findings are consistent with low endemicity settings and support the role of prenatal screening in identifying previously undiagnosed infection and enabling linkage to postpartum care. The studies reviewed showed substantial heterogeneity in prevalence estimates, reflecting differences in population characteristics, sample sizes, study periods, and methodologies. The under-representation of rural areas and the exclusion of illiterate women may have led to an underestimation of seroprevalence and should be considered when interpreting the results. Therefore, the findings are primarily generalizable to literate pregnant women attending public maternity hospitals in urban and peri‑urban áreas.

## Highlights

• Population-based study of 1202 pregnant women in Paraná, Brazil.
• Anti-HCV antibody seroprevalence was 0.75%, with no active infections detected.
• Over half of seropositive women were unaware of prior HCV exposure.
• Findings support universal prenatal HCV screening as a postpartum care opportunity, even in low-prevalence settings.
• Regional heterogeneity underscores the need for equitable screening policies in Brazil.

## Introduction

In 2016, the *World Health Organization* (WHO) highlighted that viral hepatitis is one of the main global health challenges. Estimates indicate that, by 2015, approximately 71 million people were chronically infected with the Hepatitis C Virus (HCV), which represented around 1% of the world's population at the time.^1^ Viral hepatitis is recognized as a significant cause of morbidity and mortality, mainly due to its progression to liver cirrhosis and hepatocellular carcinoma. In Brazil, it is estimated that between 1.4 and 1.7 million people have HCV, with approximately 10,000 new cases reported each year. The vast majority of those infected are unaware of their diagnosis.^2^

HCV transmission can occur through percutaneous, sexual, or vertical routes. The average rate of vertical transmission is estimated at around 5%‒6%, and can reach 8%‒15% when there is a high maternal viral load or HIV co-infection.^3^, ^4^, ^5^ This risk is strongly associated with maternal viremia, and transmission is rare in pregnant women with undetectable RNA.^6^^,^^7^ Worldwide, 1%‒8% of pregnant women have markers of HCV infection.^5^^,^^7^

In addition to the general impacts, HCV during pregnancy is associated with adverse maternal outcomes such as pre-eclampsia (Huang et al., 2016), gestational diabetes.^8^, ^9^, ^10^ and intrahepatic cholestasis.^11^ Fetal and neonatal outcomes include low birth weight, intrauterine growth restriction, prematurity.^8^^,^^9^^,^^12^ and adverse neurological events.^13^ Currently, there is no prophylaxis available for the neonate and antiviral treatment is contraindicated during pregnancy.^5^^,^^14^

Universal and early screening of pregnant women for HCV is recommended by the American College of Obstetricians and Gynecologists.^7^ especially given the increased prevalence in women of childbearing age and the lack of effective methods to prevent vertical transmission. Considering that the infection is often asymptomatic and has a silent evolution, identifying cases during prenatal care is essential for planning postpartum management and preventing future pregnancies. In this context, cross-sectional surveys are valuable tools for estimating prevalence and characterizing associated factors in specific populations, supporting public policies and control strategies.

This study is part of the national multicenter survey on the prevalence of sexually transmitted infections in Brazilian public maternity hospitals, coordinated by Wendland et al.^15^ The specific analysis of pregnant women treated in the state of Paraná is presented here, with a focus on HCV infection. The objectives were: 1) To estimate the anti-HCV antibody seroprevalence among pregnant women treated in public maternity hospitals in Paraná; and 2) To compare these findings with prevalence data obtained from pregnant women in other Brazilian states.

## Methods

This is an observational, descriptive, and cross-sectional population-based study to assess prevalence, part of the national multicenter survey on sexually transmitted infections in Brazilian public maternity hospitals, coordinated by Wendland et al.^15^ In Paraná, the survey was carried out in 14 public maternity hospitals located in different regions of the state. A detailed description of the participating maternity hospitals and the number of participants recruited at each facility is provided in Table 1. The study included pregnant women aged 16 to 49, who participated voluntarily after signing the Informed Consent Form (ICF). Post-abortion care (spontaneous or induced) and illiterate women were excluded due to their inability to read and sign the informed consent form.

**Table 1:** Maternity hospitals co-participating in the study.

Region	City	Hospital	Number of participants included per hospital
Curitiba (capital of Paraná)	Curitiba	Vitor Ferreira do Amaral Maternity	69
	Curitiba	Clinical Hospital	35
	Curitiba	Hospital do Trabalhador Hospital and Maternity	23
Metropolitan area of Curitiba	São José dos Pinhais	São José dos Pinhais Municipal Hospital And Maternity	200
	Fazenda Rio Grande	Nossa Senhora Aparecida Hospital and Maternity	147
	Campina Grande do Sul	Angelina Caron Hospital	66
	Lapa	Humberto Carrano Municipal Maternity	57
Other regions of Paraná	Apucarana	Nossa Senhora aas Graças Hospital and Maternity	173
	Cascavel	University Hospital of Western Parana	100
	Pitanga	São Vicente de Paulo Hospital	85
	Londrina	Lucilla Ballallai Municipal Maternity	68
	Santo Antônio da Platina	Norte Pioneiro Regional Hospital	63
	Cornélio Procópio	Santa Casa de Cornélio Procópio Hospital	60
	Laranjeiras do Sul	São Lucas Hospital Medical Center	56
Total			1202

Source: Prepared by the author (2024).

### *Sample*

The sample was calculated to estimate the proportion of pregnant women with hepatitis C in the state of Paraná. In 2021, 682 cases of the disease were confirmed.^16^ for a total estimated population of 11,444,380 inhabitants.^17^ which corresponds to an approximate prevalence of 0.006% (0.00006). A margin of error of 0.05% was adopted, with a 95% confidence level and an estimated finite population of 141,976 live births in that year.^18^ Based on these parameters, a minimum of 916 pregnant women were estimated to make up the study sample. The final sample included 1202 pregnant women, exceeding the minimum required.

### Considerations on representativeness

The geographical distribution of the sample was 11% from Curitiba (n = 127), 39% from the Metropolitan Region (n = 470), and 50% from other municipalities (n = 605). This proportion is close to the population distribution recorded in the 2022 Census (16%, 31%, and 53%, respectively).^17^ indicating adequate coverage of different regions of the state within the public maternity hospital network. However, the study population was restricted to literate pregnant women receiving care in public maternity hospitals, which are predominantly located in urban and peri‑urban areas. Within the category of “other municipalities”, there was a predominance of urban centers, with limited inclusion of remote rural areas. Illiterate women were excluded due to ethical requirements related to informed consent. Future studies should consider ethically approved alternatives to written informed consent, such as witnessed verbal consent and the use of thumbprints, to improve equity and representativeness.

### *Obtaining data*

Participants were randomly selected every other day by the local collection team, following the standardized protocol of the national multicenter study. The first stage consisted of filling in two structured forms: Clinical form ‒ information on prenatal care and reproductive health, obtained from the medical records and the prenatal care card; Socioeconomic and behavioral form ‒ information on socioeconomic conditions, quality of prenatal care, knowledge of Sexually Transmitted Infections (STIs), sexual behaviors and practices, applied per interviewer.

The variables related to potential risk factors for HCV were obtained using a standardized questionnaire, administered in person by previously trained interviewers. All the answers depended on the participants' self-reports and are therefore subject to memory bias and underreporting, especially for socially sensitive behaviors, possibly influenced by social desirability bias.

The second stage consisted of collecting a biological sample on filter paper (dried blood), obtained by capillary puncture. For HCV, serological screening was carried out using the ELISA method (Imunoscreen HCV ‒ SS, MBiolog Diagnósticos, Contagem, MG) at the Health Research Institute of the University of Caxias do Sul. The tests strictly followed the manufacturer's instructions, using automated equipment (Bio-Rad EVOLIS Premium System, Bio-Rad, Marnes-la-Coquette, France). The confirmatory test was carried out using viral load detection (Xpert® HCV viral load, Cepheid® PCRplus) at the Clinical Epidemiology Laboratory of the Federal University of Health Sciences in Porto Alegre.

The third stage was a literature review to identify cross-sectional studies estimating the prevalence of hepatitis C in pregnant women in Brazil. The search was carried out in July 2023 in the MEDLINE/PubMed and Virtual Health Library databases, using the MeSH terms “Hepatitis C”, “pregnant women” and “Brazil”, complemented by a manual search in the references of the articles found. The authors included cross-sectional studies with a population of pregnant women in Brazil, which clearly reported the prevalence of hepatitis C. Studies with a non-pregnant population, from other countries, or which did not explicitly report HCV prevalence were excluded.

**Fig. 1:**
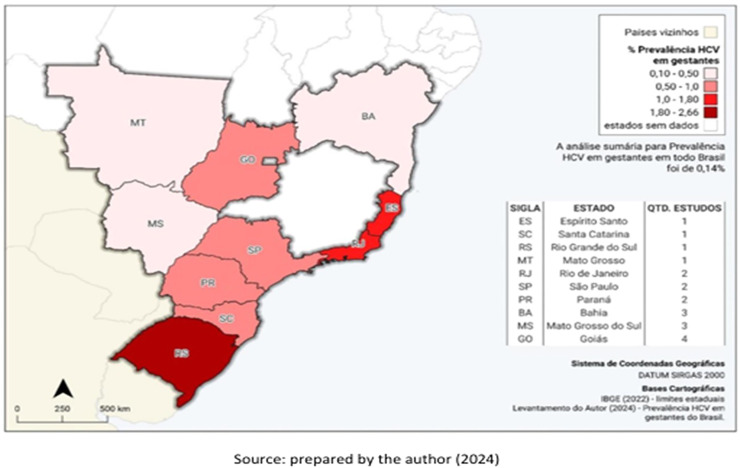
Geographic distribution of Brazilian studies on hepatitis C in pregnant women. Note: This figure illustrates only the Brazilian states in which cross-sectional studies reporting hepatitis C in pregnant women have been conducted. The prevalence values shown correspond to each individual study and are presented exclusively for descriptive contextualization. These estimates originate from studies conducted in different years, populations, and methodological designs and are therefore not directly comparable across states. No pooled prevalence, weighting, regional synthesis, or meta-analysis was performed. The absence of data in a state indicates that no eligible study was identified, rather than suggesting zero prevalence.

**Fig. 2:**
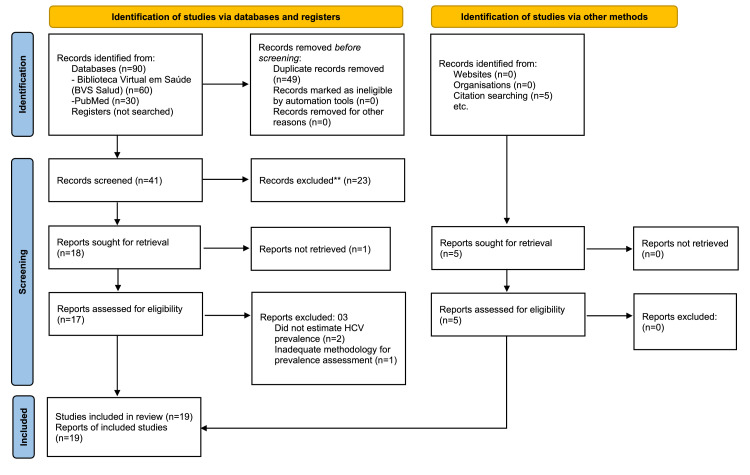
Flowchart of study selection adapted from PRISMA 2020 for a narrative review. * Consider, if feasible to do so, reporting the number of records identified from each database or register searched (rather than the total number across all databases/registers). ** If automation tools were used, indicate how many records were excluded by a human and how many were excluded by automation tools. *** This flowchart was adapted from the PRISMA 2020 statement to enhance transparency in the study selection process; however, this review did not follow a full systematic review protocol. This work is licensed under CC BY 4.0. To view a copy of this license, visit https://creativecommons.org/licenses/by/4.0/.

Source: Page MJ, et al. BMJ 2021;372:n71. doi: 10.1136/bmj.n71.

The studies included in Table 4 and Fig. 1 were obtained through a non-systematic search, with the aim of contextualizing the findings of this study. The process of identification, screening, eligibility and inclusion of the studies is described in Fig. 2, which has been adapted from the PRISMA 2020 guidelines solely for the purpose of transparently presentingthe stages of the search and selection of articles for the narrative synthesis. This was not a systematic review or formal meta-analysis. The data presented reflects a narrative synthesis of the reported prevalences, without the application of statistical weighting or formal assessment of bias, aimed at contextualizing the findings of this study.

### *Statistical analysis*

Statistical analyses were carried out using the SPSS 30.0.0 program. Categorical variables were expressed as absolute (n) and relative (%) frequencies and compared using Fisher’s exact test or the Fisher-Freeman-Halton exact test, as appropriate. To account for multiple comparisons, p-values were adjusted using the Benjamini-Hochberg false discovery rate procedure. Adjusted p-values < 0.05 were considered statistically significant.

### *Ethical aspects*

The research fully respected the ethical and legal precepts established by National Health Council Resolution 466/12. The study was approved by the Human Research Ethics Committee of the Hospital de Clínicas do Paraná Complex (CHC/UFPR), under opinion n° 5.277.188, issued on March 7, 2022. All the participants received clear information about the objectives, risks, and benefits of the research and signed the Informed Consent Form (ICF) before being included in the study.

## Results

Of the 1202 pregnant women included in the study, 470 came from the Metropolitan Region of Curitiba, 127 from the capital of Paraná and 605 from other regions of the state (Table 2). Of the total, 57.2% were over 25-years old. As for skin color, 55.2% declared themselves white. The predominant monthly family income was up to two minimum wages for 73.6% of the participants. With regard to schooling, 60.6% had completed or incomplete high school. As for marital status, 82.4% had a partner at the time of the survey (married, in a stable union, living together or not).

**Table 2:** Sociodemographic characteristics.

Variable	General (n = 1202), n (%)	Metropolitan region of Curitiba^a^ (n = 470), n (%)	Curitiba (n = 127), n (%)	Other regions in the state of Paraná (n = 605), n (%)
Age (years)				
≤ 25	514 (42.8)	193 (41.1)	63 (49.6)	258 (42.6)
> 25	688 (57.2)	277 (58.9)	64 (50.4)	347 (57.4)
Race/color				
White	663 (55.2)	279 (59.4)	68 (53.5)	316 (52.2)
Brown	424 (35.3)	151 (32.1)	43 (33.9)	230 (38)
Black	82 (6.8)	36 (7.7)	13 (10.2)	33 (5.5)
Other	14 (1.2)	2 (0.4)	2 (1.6)	10 (1.7)
Not reported	19 (1.6)	2 (0.4)	1 (0.8)	16 (2.6)
Family income				
≤1000	127 (10.6)	49 (10.4)	5 (3.9)	73 (12.1)
1001 a 3000	757 (63)	298 (63.4)	73 (57.5)	386 (63.8)
3001 a 5000	195 (16.2)	69 (14.7)	25 (19.7)	101 (16.7)
> 5000	58 (4.8)	25 (5.3)	16 (12.6)	17 (2.8)
Not reported	65 (5.4)	29 (6.2)	8 (6.3)	28 (4.6)
Education				
Elementary school complete/incomplete	288 (24)	123 (26.2)	23 (18.1)	142 (23.5)
High School complete/incomplete	728 (60.6)	280 (59.6)	79 (62.2)	369 (61)
Higher Education	167 (13.9)	63 (13.4)	25 (19.7)	79 (13.1)
Not reported	19 (1.6)	4 (0.9)	‒ (‒)	15 (2.5)
Marital status				
Single	177 (14.7)	94 (20)	22 (17.3)	61 (10.1)
Has a partner, but they live in separate houses/Lives currently with partner/ Married	990 (82.4)	367 (78.1)	102 (80.3)	521 (86.1)
Separated or divorced/Widowed	14 (1.2)	4 (0.9)	3 (2.4)	7 (1.2)
Not reported	21 (1.7)	5 (1.1)	‒ (‒)	16 (2.6)
Number of partners				
1	246 (20.5)	75 (16)	21 (16.5)	150 (24.8)
1‒3	328 (27.3)	117 (24.9)	29 (22.8)	182 (30.1)
> 3	372 (30.9)	150 (31.9)	55 (43.3)	167 (27.6)
Not reported	256 (21.3)	128 (27.2)	22 (17.3)	106 (17.5)
Number of pregnancies				
1	389 (32.4)	162 (34.5)	54 (42.5)	173 (28.6)
> 1	779 (64.8)	302 (64.3)	73 (57.5)	404 (66.8)
Not reported	34 (2.8)	6 (1.3)	‒ (‒)	28 (4.6)
Delivery route				
Vaginal	578 (48.1)	187 (39.8)	44 (34.6)	243 (40.2)
Cesarean section	609 (50.7)	283 (60.2)	83 (65.4)	347 (57.4)
Not reported	15 (1.2)	‒ (‒)	‒ (‒)	15 (2.5)
Have you used or tried drugs in your life?				
Yes	242 (20.1)	92 (19.6)	43 (33.9)	107 (17.7)
No	915 (76.1)	364 (77.4)	84 (66.1)	467 (77.2)
Not reported	45 (3.7)	14 (3)	‒ (‒)	31 (5.1)
Gestational age 1st prenatal visit (weeks)				
≤ 12	728 (60.6)	278 (59.1)	77 (60.6)	373 (61.7)
>12	236 (19.6)	92 (19.6)	25 (19.7)	118 (19.5)
Not reported	238 (19.8)	100 (21.3)	25 (19.7)	114 (18.8)
Planned pregnancy				
Yes	521 (43.3)	208 (44.3)	46 (36.2)	267 (44.1)
No	663 (55.2)	260 (55.3)	81 (63.8)	322 (53.2)
Not reported	18 (1.5)	2 (0.4)	0 (0)	16 (2.6)
Have you been vaccinated for Hepatitis B?				
Yes (at least 1 dose)	940 (78.2)	362 (77)	81 (63.8)	497 (82.1)
No	94 (7.8)	54 (11.5)	14 (11)	26 (4.3)
Not reported	168 (14)	54 (11.5)	32 (25.2)	82 (13.6)

a Without considering Curitiba. Source: Prepared by the author (2024).

With regard to prenatal care and obstetric history, 64.8% were multiparous. Considering gestational age at the first appointment ‒ an indicator of prenatal care quality ‒ 60.6% started follow-up at up to 12-weeks. The pregnancy was planned in 43.3% of cases. As for the outcome by route of delivery, caesarean section was performed in just over half of the cases (50.7%). Table 2 summarizes the main socio-demographic characteristics of the study population.

Nine participants were anti-HCV seropositive, yielding a seroprevalence of 0.75% (95% CI 0.34%‒1.40%). All reactive cases had undetectable HCV RNA, indicating no active infection in the study population. Among these nine pregnant women, 5 (55%) reported never having been previously diagnosed with HCV, while four were unable to provide information (Table 3).

**Table 3:** Pregnant women infected with HCV.

Variables	Reagents (sorting) (n = 9), n (%)	No reactive or indeterminate (screening) (n = 1193), n (%)	p-value without correction	p-value with correction
Region			0.701^a^	1.0^c^
Metropolitan area of Curitiba	3 (33.3)	467 (39.1)		
Curitiba	0 (0)	127 (10.6)		
Other regions of Paraná	6 (66.7)	599 (50.2)		
Age (years)			0.043^b^	0.387^c^
≤ 25	7 (77.8)	507 (42.5)		
> 25	2 (22.2)	686 (57.5)		
Race/color			1^a^	1.0^c^
White	6 (66.7)	657 (55.1)		
Brown	3 (33.3)	421 (35.3)		
Black	0 (0)	82 (6.9)		
Other	0 (0)	14 (1.2)		
Not reported	0 (0)	19 (1.6)		
Family income			0.834^a^	1.0^c^
≤ 1000	1 (11.1)	126 (10.6)		
1001 a 3000	6 (66.7)	751 (63)		
3001 a 5000	1 (11.1)	194 (16.3)		
> 5000	0 (0)	58 (4.9)		
Not reported	1 (11.1)	64 (5.4)		
Education			0.900^a^	1.0^c^
Elementary school complete/incomplete	3 (33.3)	285 (23.9)		
High School complete/incomplete	5 (55.6)	723 (60.6)		
Higher Education	1 (11.1)	166 (13.9)		
Not reported	0 (0)	19 (1.6)		
Marital status			0.095^a^	0.427^c^
Single	4 (44.4)	173 (14.5)		
Has a partner, but they live in separate houses/Lives currently with partner/ Married	5 (55.6)	985 (82.6)		
Separated or divorced/Widowed	0 (0)	14 (1.2)		
Not reported	0 (0)	21 (1.8)		
Number of partners			0.536^a^	1.0^c^
1	2 (22.2)	244 (20.5)		
1‒3	4 (44.4)	324 (27.2)		
> 3	1 (11.1)	371 (31.1)		
Not reported	2 (22.2)	254 (21.3)		
Have you been vaccinated for Hepatitis B?			0.682^a^	1.0^c^
Yes (at least 1 dose)	7 (77.8)	933 (78.2)		
No	0 (0)	94 (7.9)		
Not reported	2 (22.2)	166 (13.9)		
Has a health professional ever told you that you had Hepatitis C?			0.513^a^	1.0^c^
Yes	0 (0)	1 (0.1)		
Not reported	4 (44.4)	683 (57.3)		
No	5 (55.6)	509 (42.7)		

a Significance of Fisher-Freeman-Halton test (p < 0.05).

b Significance of Fisher's exact test (p < 0.05).

c Significance adjusted by Benjamini-Hochberg correction for multiple testing (nine tests), p < 0.05. Statistical significance should be interpreted based on the adjusted p-values after Benjamini-Hochberg correction. Source: prepared by the author (2024).

Age-stratified analyses did not identify a statistically significant association between maternal age and anti-HCV seropositivity after correction for multiple testing. Age was initially explored using different categorizations (≤ 20, 20–30, and > 30-years; ≤ 25 vs > 25-years). In unadjusted analyses, a higher proportion of reactive cases was observed among women aged ≤ 20-years (χ² = 10.1; df = 2; p = 0.006; Fisher-Freeman-Halton exact test = 0.017), and a similar distribution was noted when using a 25-year cut-off. However, after adjustment for multiple comparisons using the Benjamini-Hochberg procedure, the association with age was no longer statistically significant. No other sociodemographic or obstetric variables showed significant associations with anti-HCV seropositivity after correction (Table 3).

As for the literature review, fourteen studies were identified by the eligibility criteria, and five by manual search, totaling 19 studies (Fig. 2). Prevalence estimates reported in these studies ranged from 0.06% to 2.66%, reflecting substantial heterogeneity in sample size, study period, and methodology (Table 4). The geographic distribution of these studies is illustrated in Fig. 1, which indicates the states where prevalence data are available.

**Table 4:** HCV prevalence studies in pregnant women in Brazil.

Hepatitis C Study	Year	Location (city‒state)	Sample	HCV+	% PHCV
Lima, LH (40).^23^	1999	Vitória‒ES	444	6	1.35%
Martins (46).^37^	1990‒1992	Goiania‒GO	1273	22	1.73%
Lima, MP (45).^36^	1994‒1998	Campinas‒SP	6995	54	0.77%
Reiche (24).^35^	1996‒1999	Londrina‒PR	1006	8	0.80%
Peixoto (41).^39^	1998‒1999	Porto Alegre‒RS	1090	29	2.66%
Orione (60).^40^	2002	Cuiabá‒MT	1607	6	0.37%
Figueiró Filho (19).^41^	2002‒2003	Campo Grande‒MS	32,512	30	0.09%
Gardenal (39).^24^	2002‒2005	Campo Grande‒MS	31,187	58	0.19%
Gomes Filho (57).^42^	2003‒2009	GO	348,037	334	0.10%
Costa ZB (44).^43^	2004‒2005	Goiania‒GO	28,561	65	0.23%
Pinto (43).^44^	2005‒2007	MS	115,386	124	0.11%
Fernandes (16).^45^	2005‒2009	Catalão‒GO	1641	2	0.12%
Barros (13).^25^	2006‒2013	Niterói‒RJ	560	7	1.25%
Gonçalves (53).^46^	2007	SJRP‒SP	545	4	0.73%
Vilte (59).^47^	2008‒2012	Niterói‒RJ	922	15	1.63%
Passini (42).^38^	2009‒2011	Salvador‒BA	3049	12	0.39%
Batistão (18).^48^	2014‒2016	Tubarão‒SC	243	2	0.82%
Freire (14).^26^	2014‒2017	Salvador‒BA	16.639	10	0.06%
Vargas (36).^49^	2016‒2017	Salvador‒BA	2099	6	0.29%
Aquery et al. (Present Study)	2022‒2023	PR	1202	9	0.75%

SJRP, São José do Rio Preto; HCV+, Positive HCV cases; % PHCV, HCV Prevalence rate.

Abbreviation code of Brazilian states: BA, Bahia; ES, Espírito Santo; GO, Goiás; MT, Mato Grosso; MS, Mato Grosso do Sul; PR, Paraná; RJ, Rio de Janeiro; RS, Rio Grande do Sul; SC, Santa Catarina; SP, São Paulo.

Note: The included studies are highly heterogeneous with respect to study period, sample size, geographic setting, and methodological approaches; therefore, no pooled or aggregated prevalence estimate was calculated.

Source: Prepared by the author (2024).

## Discussion

This study showed an overall anti-HCV antibody seroprevalence of 0.75% among pregnant women, a figure similar to that observed in India (0.6%).^19^ and lower than the r reported in countries such as the United States and Europe (1%‒2%).^20^ Pakistan (1.8%).^21^ and Poland (2%).^22^ In developing countries, prevalence rates can reach up to 8%.^20^ In Brazil, data remains scarce and heterogeneous.^23^, ^24^, ^25^, ^26^

In the present sample, all pregnant women with positive serology had undetectable viremia, indicating no active infection at the time of testing. The discrepancy observed between anti-HCV seropositivity (0.75%), and the absence of viral RNA detection (0%) can be explained by different mechanisms. One is the spontaneous resolution of the infection, which occurs in around 15% to 45% of cases, leading to the persistence of antibodies even in the absence of viral replication.^27^ Another possibility is the occurrence of false-positive results in serological tests, especially in low-prevalence populations, even if third-generation assays are used.^28^ In addition, the viral load may be intermittently below the detection limit at the time of collection.^27^ or the pregnant woman may have received previous treatment with antivirals, resulting in sustained virological cure and maintenance of residual seropositivity.^28^ Although less frequent, nonspecific serological responses resulting from cross-immunity with other flaviviruses can also occur.^29^

This finding is relevant from a clinical and epidemiological point of view, since vertical transmission of HCV occurs almost exclusively in cases with detectable viremia.^4^^,^^6^ Although in this cohort the immediate risk of transmission was non-existent, the detection of anti-HCV antibodies indicates previous exposure and justifies referral for confirmatory diagnosis and postpartum treatment with Direct-Acting Antivirals (DAAs), whose cure rate exceeds 95% in chronic cases.^30^ Although the immediate risk of vertical transmission was non-existent in this cohort, prenatal screening remains relevant primarily as an opportunity to identify past or undiagnosed infection and ensure linkage to postpartum care, rather than reflecting a high burden of active disease. International guidelines, such as those of the AASLD-IDSA.^30^ recommend testing all pregnant women, taking advantage of prenatal care as a unique diagnostic opportunity for women who often have little or no previous contact with health services.^4^ Chaillon et al.^31^ e Tasillo et al.^32^ show that, even in populations with a prevalence of <0.1%, universal screening is cost-effective, reducing total costs by avoiding complications such as cirrhosis, hepatocellular carcinoma and liver transplantation, as well as interrupting future transmission chains ‒ a scenario that is in line with that found in this study.

However, in Brazil, the implementation of this strategy is uneven: despite the Ministry of Health recommending universal testing for hepatitis C in pregnant women since 2020, the test is not part of the mandatory screening list in many regions.^2^ In Paraná, even with high prenatal care coverage ‒ 86.10% of pregnant women had ≥ 7 appointments.^33^ ‒ HCV testing is not systematically offered. This may partly explain the fact that at least 55% of HCV-positive pregnant women in the study were unaware of their infection. This percentage may be even higher, considering that in four cases it was not possible to obtain this information, which suggests a combination of flaws in screening protocols during prenatal care and limitations in self-reporting. In addition, a lack of prior knowledge may be associated with poor understanding of the results and communication barriers, especially in populations with lower health literacy. In this study, 60.6% of the participants had up to high school education, and 73.6% lived on a monthly family income of up to two minimum wages ‒ indicators which, in the Brazilian context, are often associated with greater social vulnerability.

From a public health perspective, the absence of detectable viremia among 1202 pregnant women indicates an extremely low prevalence of active hepatitis C infection in this population. In such a context, consideration of the balance between universal and targeted screening strategies is warranted, particularly in relation to the efficient allocation of healthcare resources. While universal prenatal screening is recommended by international guidelines and supported by cost-effectiveness analyses even in low-prevalence settings, the near-zero observed viremia may inform local health service planning in Paraná. This may include prioritizing testing among higher-risk subgroups or integrating HCV screening within broader prenatal infectious disease panels. Therefore, in this setting, prenatal HCV screening should be viewed less as a response to an immediate transmission risk and more as a long-term public health strategy aimed at identifying previously undiagnosed infection and ensuring appropriate linkage to postpartum care.

In this study, the sample was geographically distributed across different regions of Paraná within the public maternity hospital network. However, the study population was restricted to literate pregnant women receiving care in public maternity hospitals, which are predominantly located in urban and peri‑urban areas. Within this context, heterogeneity in HCV seroprevalence was observed, with rates of 0% in the capital, 0.64% in the Metropolitan Region, and 1.0% in other municipalities. A similar pattern was described in the ecological study by Silva et al.^34^ conducted in the general population of Paraná, which identified a heterogeneous distribution of hepatitis C across health regions. Such variation is explained by contextual and systemic factors, including differences in healthcare organization, access to diagnostic services, local surveillance capacity, and sociodemographic vulnerability across regions, rather than by individual-level characteristics.

The percentage of pregnant women with anti-HCV seropositivity found in this study was 0.75%, within the range reported in previous cross-sectional studies of pregnant women in Brazil, which varied from 0.06% to 2.66%, reflecting substantial heterogeneity in study period, sample size, and methodology. Despite these differences, the present findings are consistent with the only previous study conducted among pregnant women in Paraná, which reported a prevalence of 0.80% in Londrina.^35^ Data from the National Health Survey (PNS 2020) indicate prevalence rates of between 0.2% and 0.8% in the 15‒49 age group ‒ corresponding to the reproductive group ‒ and 1.5% in the general population.^2^ Taken together, these findings highlight the wide variability of anti-HCV seroprevalence reported among pregnant women in Brazil and underscore the influence of study design, population characteristics, and healthcare context on prevalence estimates. Such differences may reflect regional and contextual factors, including access to health services, availability of HCV testing, socioeconomic and educational profiles, and the distribution of risk factors across different healthcare settings. In this study, age-stratified analyses were conducted as an exploratory approach. Although unadjusted analyses suggested a higher proportion of anti-HCV seropositivity among younger pregnant women using different age categorizations, no statistically significant association between age and anti-HCV seropositivity was observed after adjustment for multiple testing. Therefore, age cannot be considered a risk factor in this cohort. Any apparent differences across age groups should be interpreted with caution and likely reflect random variation due to the small number of positive cases rather than a true epidemiological pattern.

Other traditional risk factors ‒ blood transfusions.^24^^,^^36^^,^^37^ tattoos.^36^, ^37^, ^38^ and the use of injectable or inhalable drugs.^36^^,^^38^ ‒ showed no significant association in this sample. This absence should be interpreted with caution, since the data were obtained by self-report and may have been influenced by social desirability bias or the omission of stigmatized behaviours.

Despite the limitations ‒ such as the exclusion of illiterate women, the small number of positive cases, and the biases inherent in self-reporting ‒ this study provides relevant data on hepatitis C in pregnant women in Paraná. The prevalence observed, which is higher than the national average, shows that the state's reality may be underestimated in broader surveys. In addition, the heterogeneity between the regions evaluated, possibly related to inequalities in access to healthcare and different sociodemographic profiles, reinforces the need for uniform screening strategies. These findings support universal prenatal screening as a strategy for early identification of previous or undiagnosed infection and for linkage to postpartum care and treatment, even in settings with very low or absent active infection. In this context, screening should be viewed not primarily as a response to immediate transmission risk, but as a public health opportunity for long-term disease control. The systematic incorporation of HCV testing into prenatal care protocols, therefore, represents a strategic opportunity to advance towards the elimination of HCV as a public health problem in Brazil.

## Data availability

All data generated or analyzed during this study are included in this published article. Additional data used for comparison of prevalence were obtained from publicly available databases, including the Ministry of Health (DATASUS/SINAN) and the Brazilian Institute of Geography and Statistics (IBGE), as cited in the References section.

## Authors’ contributions

Gabriele Aquery: Conceptualization; methodology; investigation; data curation; formal analysis; writing-original draft; writing-review & editing.

Newton S. de Carvalho: Supervision; conceptualization; methodology; project administration; writing-review & editing.

## Declaration of competing interest

The authors declare no conflicts of interest.
